# Supplementary figures and images for: Diagnostic performance of abbreviated non-contrast liver MRI for detecting synchronous colorectal liver metastases
Source: PLoS One. 2026 May 13;21(5):e0348972. doi: 10.1371/journal.pone.0348972 (PMC13170879; doi:10.1371/journal.pone.0348972)

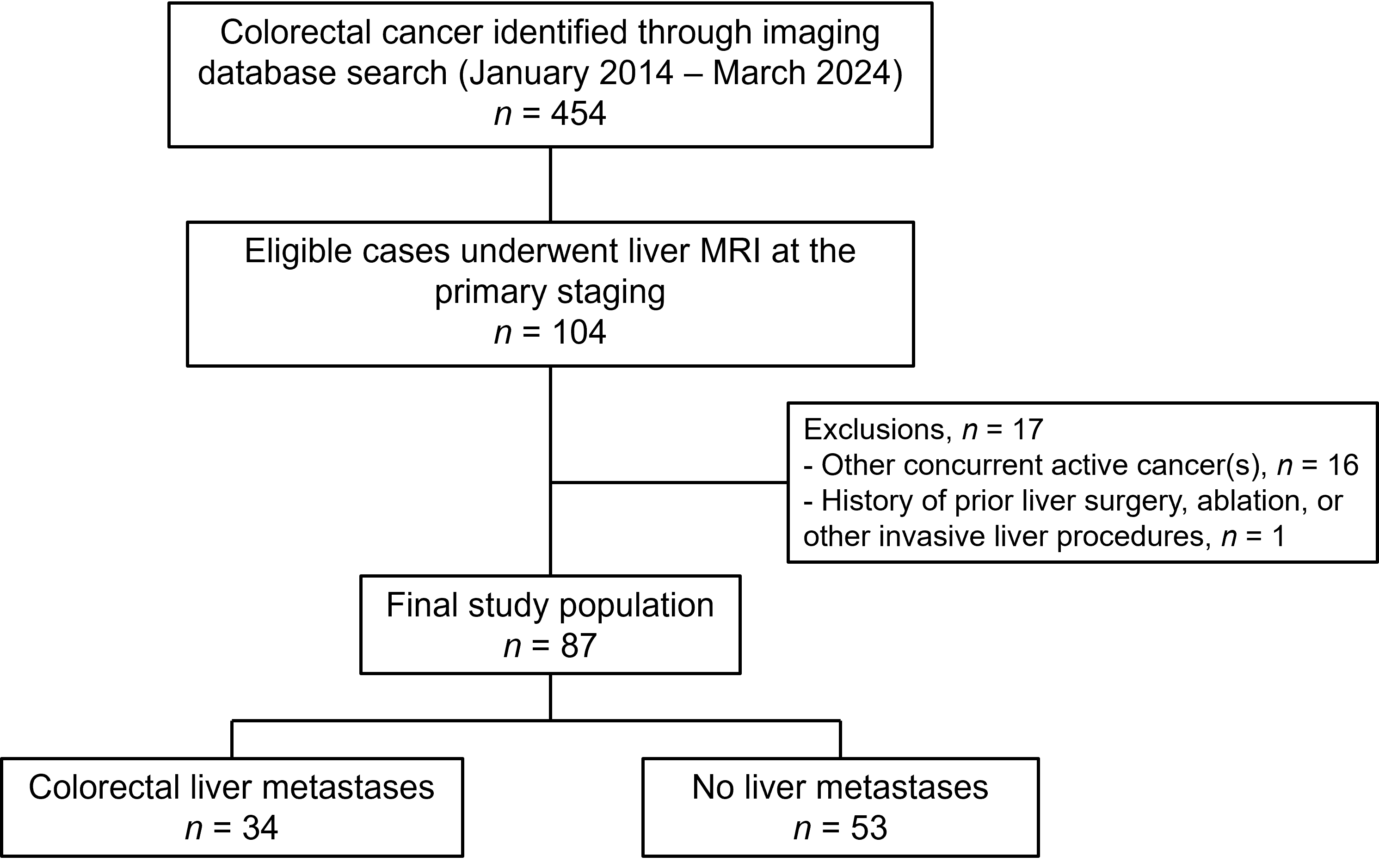

Supplement: S1 Fig — A total of 454 patients with CRC were identified through an imaging database search. Among them, 104 patients underwent liver MRI at primary staging. After excluding 17 patients (16 with other active malignancies and one with prior liver surgery or invasive liver procedures), 87 patients were included in the final analysis. (TIF) [file pone.0348972.s001.tif]

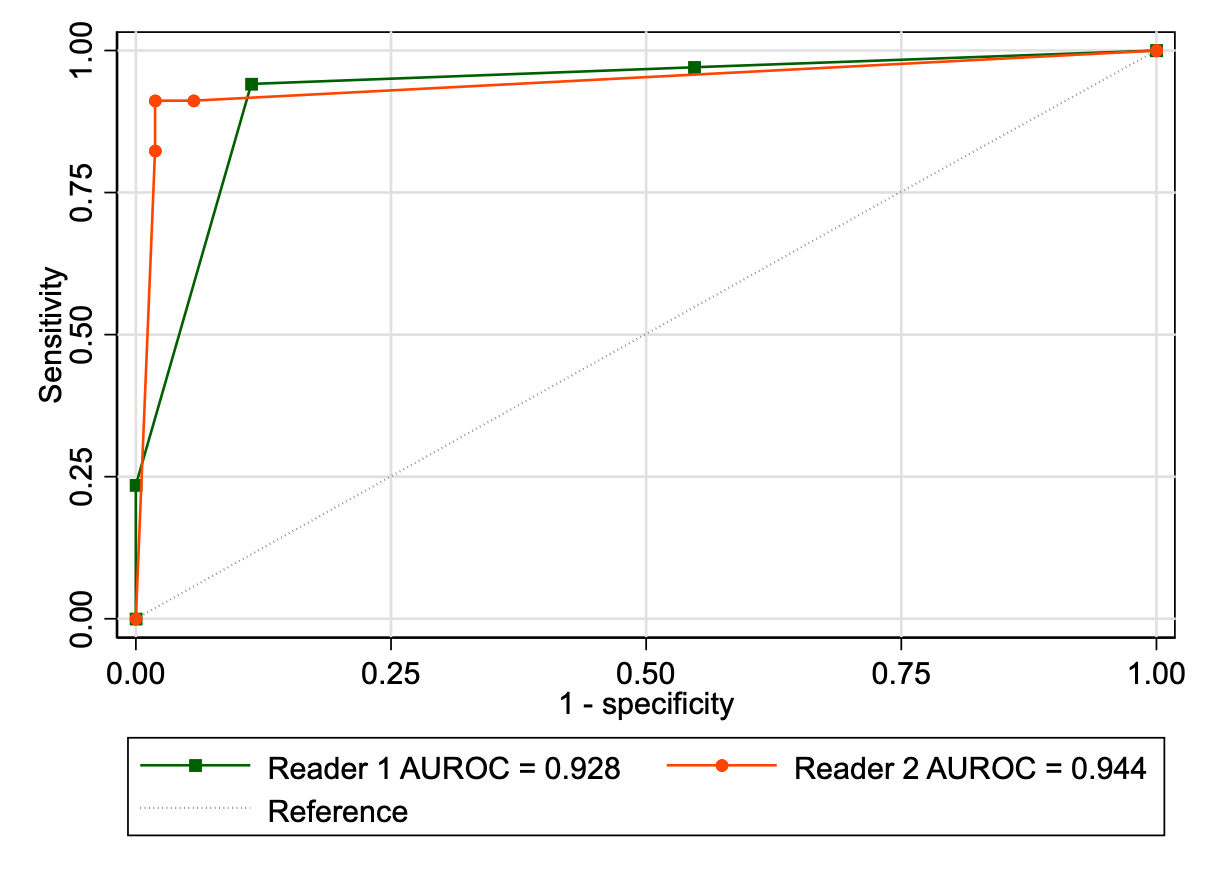

Supplement: S2 Fig — ROC curves were generated using the 4-point diagnostic confidence scores for each reader. (TIF) [file pone.0348972.s002.tif]
