# Supplementary material for: Diagnostic performance of abbreviated non-contrast liver MRI for detecting synchronous colorectal liver metastases
Source: PLoS One. 2026 May 13;21(5):e0348972. doi: 10.1371/journal.pone.0348972 (PMC13170879; doi:10.1371/journal.pone.0348972)
Supplement: S1 Table — Two-by-two tables showing classification of metastasis and non-metastasis by each reader compared with the reference standard. (DOCX) [file pone.0348972.s003.docx]

**S1 Table.** Contingency tables showing the classification of metastasis and non-metastasis by each reader compared with the reference standard (contrast-enhanced MRI).

(A) Reader 1

|  | Reference standard | |  |
| --- | --- | --- | --- |
| MRI results | CRLM present | No CRLM | Total |
| Positive | 32 | 6 | 38 |
| Negative | 2 | 47 | 49 |
| Total | 34 | 53 | 87 |

(B) Reader 2

|  | Reference standard | |  |
| --- | --- | --- | --- |
| MRI results | CRLM present | No CRLM | Total |
| Positive | 31 | 2 | 33 |
| Negative | 3 | 51 | 54 |
| Total | 34 | 53 | 87 |
