# Supplementary material for: Diagnostic performance of abbreviated non-contrast liver MRI for detecting synchronous colorectal liver metastases
Source: PLoS One. 2026 May 13;21(5):e0348972. doi: 10.1371/journal.pone.0348972 (PMC13170879; doi:10.1371/journal.pone.0348972)
Supplement: S2 Table — Detailed description of FP and FN cases for each reader, including lesion characteristics and final diagnosis. (DOCX) [file pone.0348972.s004.docx]

**S2 Table.** Detailed analysis of false-positive (FP) and false-negative (FN) cases.

| Case | Reader(s) | Error type | Confidence score | Reference result | No. of lesions | Explanation | Basis of confirmation |
| --- | --- | --- | --- | --- | --- | --- | --- |
| 1 | R1, R2 | FN | 0, 0 | CRLM present | 1 | Very small metastasis adjacent to a tiny cyst | Resection histology |
| 2 | R2 | FN | 0 | CRLM present | 2 | Very small metastases | Enhancement; core needle biopsy before ablation |
| 3 | R1, R2 | FN | 1, 0 | CRLM present | 1 | Small lesion in caudate lobe (Fig 6) | Enhancement; follow-up showed progression |
| 4 | R1 | FP | 2 | No CRLM | 0 | Small cyst with thin septa and mild DWI hyperintensity | No enhancement; follow-up negative |
| 5 | R1 | FP | 2 | No CRLM | 0 | Multiple cysts with mild restricted diffusion | No enhancement; follow-up negative |
| 6 | R1, R2 | FP | 2, 3 | No CRLM | 0 | Restricted diffusion without contrast correlation | Hyperenhancement with washout; elevated AFP (HCC, Fig 5) |
| 7 | R1, R2 | FP | 2, 3 | No CRLM | 0 | Restricted diffusion | Tract-like lesion with eosinophilia (Fig 4) |
| 8 | R1 | FP | 2 | No CRLM | 0 | Very small lesion with mild restricted diffusion | Cyst; no enhancement |
| 9 | R1 | FP | 2 | No CRLM | 0 | Small lesion with mild restricted diffusion | Cyst; no enhancement (Fig 3) |
