# Supplementary material for: Diagnostic performance of abbreviated non-contrast liver MRI for detecting synchronous colorectal liver metastases
Source: PLoS One. 2026 May 13;21(5):e0348972. doi: 10.1371/journal.pone.0348972 (PMC13170879; doi:10.1371/journal.pone.0348972)
Supplement: Attachment — Submitted filename: Response to the Editor and Reviewers.docx [file pone.0348972.s007.docx]

Dear Editor and Reviewers,

We sincerely thank you for your careful evaluation of our manuscript and for the constructive and insightful comments. We greatly appreciate the time and effort invested in improving our work. We have thoroughly revised the manuscript in accordance with all comments and believe it has been substantially strengthened in clarity, methodological transparency, and clinical relevance.

Below, we provide a point-by-point response to each comment.

**Reviewer #1**

We are grateful for the reviewer’s positive assessment of our study and for the detailed suggestions.

**1. Statistical Analysis (Section 2.4)**

**Response:** We thank the reviewer for this important comment. We have revised Section 2.4 to improve clarity and explicitly describe the analytical approach. We now clearly state that:

- The 4-point diagnostic confidence score (0–3) was used as an ordinal variable for ROC analysis
- AUROC reflects overall discriminatory performance
- Sensitivity and specificity were calculated at a predefined clinically relevant threshold (scores 2–3 vs. 0–1)

This revision clarifies the relationship between ordinal scoring and binary clinical decision-making.

**2. AUROC vs diagnostic accuracy**

**Response:** We agree with the reviewer and have revised the manuscript to ensure conceptual clarity. The term “diagnostic accuracy” is now used only for threshold-based metrics, while AUROC is consistently described as a measure of discriminatory performance.

**3. Interobserver Agreement**

**Response:** We appreciate this suggestion. We have revised the analysis to use **quadratic weighted kappa**, which better reflects the ordinal nature of the 4-point confidence scale. This change has been incorporated into the Methods and Results sections.

**4. False-positive and false-negative findings**

**Response:** We have added a detailed summary of false-positive and false-negative cases in **Supplementary Table S2**, including the number of cases per reader and classification based on imaging and final diagnosis. Relevant examples are illustrated in Figures 3–6. This improves transparency and aligns with STARD recommendations.

**5. AI / Radiomics discussion**

**Response:** We agree that the previous statement was overly generic. We have revised the Discussion to de-emphasize AI/radiomics and instead highlight protocol optimization and quality assurance, particularly for DWI, as more immediate and clinically applicable strategies for improving diagnostic performance in abbreviated MRI protocols.

**6. DWI speculation**

**Response:** We have removed speculative explanations regarding the biological mechanisms of diffusion restriction and now present these findings as observed imaging characteristics.

**7. Predictors of false-negative cases**

**Response:** We have revised the text to clarify that these statements are hypothesis-generating and based on prior literature, rather than derived from our dataset.

**8. Terminology in Discussion**

**Response:** We have revised the Discussion to clearly distinguish between discriminatory performance (AUROC) and threshold-based diagnostic metrics, improving conceptual precision.

**9. Prevalence and performance metrics**

**Response:** We appreciate this important clarification. The relevant paragraph has been revised to state that sensitivity, specificity, and AUROC are independent of disease prevalence, while accuracy is prevalence-dependent.

**10. Imaging protocol description**

**Response:** We have revised this section to clearly specify the use of fat-suppressed T2-weighted sequences and to simplify the description, focusing on clinically relevant aspects of lesion detection.

**11. Limitations**

**Response:** We have expanded the limitations section to include:

- Broader implications of the retrospective design (including selection bias and variability in imaging conditions)
- The post hoc derivation of the abbreviated protocol
- Scanner and vendor heterogeneity, acknowledging both its potential impact on internal validity and its contribution to real-world generalizability

**12. Conclusion**

**Response:** We have revised the conclusion to:

- Use more balanced wording (“good to excellent diagnostic performance”)
- Specify applicability in selected clinical scenarios
- Replace the term “impractical” with “not feasible in resource-limited settings.”

**Reviewer #2**

We thank the reviewer for highlighting important aspects regarding novelty and clarity.

**1. Novelty of the study**

**Response:** We have revised both the Introduction and Discussion to better emphasize the novelty of our work. In contrast to prior studies that primarily employed lesion-based analyses or protocol comparisons, our study uses a patient-based approach in a real-world staging context, reflecting the clinically relevant decision of determining whether liver metastasis is present.

**2. Mucinous colorectal cancer**

**Response:** We have added a statement in the Discussion noting that mucinous tumors may present diagnostic challenges due to overlapping imaging features with cystic lesions. However, no dedicated subgroup analysis was performed, and this remains an area for future investigation.

**3. Abstract wording**

**Response:** The abstract has been revised to clearly state the study aim and align with the Introduction.

**4. Study period**

**Response:** The study period (January 2014 to March 2024) has been added to the abstract.

**5. Terminology (“kidney issues”)**

**Response:** We have replaced this wording with “renal impairment” for clarity and precision.

**6. Hypothesis and aims**

**Response:** We have repositioned and expanded the hypothesis to include both primary and secondary objectives, and placed the aims in the final paragraph of the Introduction.

**7. Table 1 (p-values)**

**Response:** We agree and have removed *p*-values from Table 1. Patient characteristics are now presented descriptively.

**8. Rounding of numbers**

**Response:** We have revised numerical values to improve readability and avoid unnecessary precision.

**9. Additional reliability metrics**

**Response:** We clarified that weighted kappa was the primary metric, and additional agreement measures were included as supportive information.

**10. Figure correction**

**Response:** The arrow placement in Figure 3 has been corrected. I’m so appreciative of your help in correcting this radiologist-matter detail.

We sincerely thank the Editor and Reviewers again for their valuable feedback. We believe the revisions have substantially improved the manuscript and we hope it is now suitable for publication.

Sincerely,

Puwitch Charoenchue, M.D., Ph.D.

On behalf of all co-authors

24 March 2026
